# Provisioning ecotourism does not increase tiger shark site fidelity

**DOI:** 10.1038/s41598-023-34446-8

**Published:** 2023-05-13

**Authors:** Clémentine Séguigne, Michel Bègue, Carl Meyer, Johann Mourier, Éric Clua

**Affiliations:** 1 PSL Université Paris: EPHE-UPVD-CNRS, USR 3278 CRIOBE, BP 1013, 98729 Papetoai, Moorea French Polynesia; 2 Laboratoire d’Excellence “CORAIL”, 98729 Papetoai, Moorea French Polynesia; 3 Hawai’i Institute of Marine Biology, University of Hawai’i at Mānoa, P. O. Box 1346, Kaneohe, HI USA; 4 MARBEC, Univ Montpellier, CNRS, Ifremer, IRD, Sète, France

**Keywords:** Behavioural ecology, Tropical ecology

## Abstract

A perennial criticism of provisioning ecotourism is that it alters the natural behavior and ecology of the target species by providing an artificial food source. Here we evaluate its impact on the long-term site fidelity patterns of tiger sharks in French Polynesia. We hypothesized that a significant impact of provisioning would lead to (1) increases in individual site fidelity over time, and (2) an increase in the number of resident individuals over time. Of 53 individuals photo-identified and monitored during > 500 dives over five years, 10 individuals accounted for > 75% of all sightings, whereas 35 sharks were sighted very infrequently. Even the most frequently observed tiger sharks exhibited overall low fidelity at the site and showed no increase in site fidelity over time. Furthermore, the number of tiger sharks sighted during each dive did not increase. The observed patterns of tiger shark sightings were best explained by natural movements, including general roaming within home ranges along the coastline and seasonal migrations. Despite the apparent lack of impact of provisioning ecotourism on tiger shark ecology in Tahitian waters, it would be prudent to implement a strict code of conduct during any future provisioning activities to maximize the safety of participants and animals involved.

## Introduction

The tiger shark *Galeocerdo cuvier* (Péron & Lesueur 1822) is a large (up to 550 cm Total Length)^1^ apex predator in tropical and temperate waters^2^. Although routinely present in coastal shelf, reef and slope habitats^2^, tiger sharks are typically rarely encountered by scuba divers in the wild, excepted under baited conditions^2,3^. This species is currently near threatened^2^ with population declines evident in several locations including Northeast Australia^4^ and the Arabian Seas Region^5^. Their large size and reputation as a potentially dangerous apex predator make tiger sharks a popular target species for shark ecotourism in locations such as Fiji, South Africa and the Bahamas, where in the latter it has contributed to a shark watching industry that has generated $800 million over a 20 years period^6–8^. The economic viability of shark tourism depends on predictable and sustainable sightings of the target species^9^ which in the case of tiger sharks typically requires the use of attractants ranging from simple olfactory stimulus (chumming) to active feeding of individuals present in the area^10^.

Shark feeding for ecotourism purposes is a controversial activity^11,12^. A primary concern is that provisioning may teach sharks to associate humans with food and thereby increase the risk of shark bites^13,14^. However, to date no demonstrable increase in shark bites has been observed at or around provisioning sites many of which have shark feeding regulations that strictly limit the amount of food that can be consumed^15–17^. A further criticism is that artificial provisioning (i.e. humans deliberately feeding wildlife) alters the natural ecology and behavior of the target species by increasing site fidelity (i.e. time spent at provisioning sites) at the expense of wider-ranging movements^18,19^. This effect is inherently difficult to assess because shark site fidelity naturally varies both inter- and intra-specifically^15,20,21^. For example, significant intraspecific variability in site fidelity is naturally present in silky sharks *Carcharhinus falciformis* (Bibron 1839)^22^, sicklefin lemon sharks *Negaprion acutidens* (Rüppel 1837)^23^, and bull sharks *Carcharhinus leucas* (Valenciennes in Müller & Henle 1839)^24^ with some individuals showing high fidelity at specific sites while others are only occasional transient visitors. The limited evidence available to date suggests that tiger shark movement patterns are not significantly affected by provisioning^25^. Despite these inherent challenges, it is important for us to understand how provisioning activities impact shark behavior and ecology in order to effectively manage shark ecotourism activities to ensure adequate protection of these iconic species^10,26^.

The major concerns surrounding provisioning ecotourism are particularly pertinent to tiger sharks because they are one of the three main shark species routinely implicated in unprovoked bites on humans (second only to white sharks *Carcharodon carcharias* (Linnaeus 1758))^27^ and show considerable natural variability in movement patterns, often showing fidelity to a specific “home” island, but also able to roam thousands of kilometers into open-ocean^28^. Tiger sharks variously show high site-fidelity to core use areas (CUA) and wide-ranging movements in coastal habitats, and wide-ranging movements and multi-month residence times in open-ocean in both the Indo-Pacific and Atlantic regions^29–32^. Seasonal migrations by tiger sharks have been documented in the Atlantic, Indian and Pacific Oceans^31,33,34^, and appear to be linked to sea surface temperature (SST), especially in more temperate regions toward the latitudinal limits of tiger shark distribution^31,34^. Tiger sharks occur in SSTs ranging from 18 to 33 °C but coastal abundance and swimming performance of tiger sharks are both highest at ~ 22 °C, suggesting thermal constraints on performance may regulate this species’ distribution^35–37^.

Tiger sharks were a focal species at the “Vallée Blanche” (VB) shark ecotourism dive site in Tahiti (French Polynesia) where shark provisioning activities occurred from 2011 until October 2017 when shark feeding was officially banned (Law of the Country 2017–25). The regular ecotourism activities at the VB site from October 7, 2012 to October 9, 2017 provided an opportunity to study the long-term impacts of provisioning on tiger shark abundance and site fidelity. We hypothesized that an impact of provisioning on tiger shark movement patterns would result in (i) increased fidelity of individual tiger sharks over time (i.e. visiting on more days and being present for longer periods), and (ii) an increase in the number of individuals observed per dive over time.

## Results

### Tiger shark relative abundance

A total of 1027 tiger shark sightings were recorded at the VB site during 544 dives conducted between 7 October 2012 and 9 October 2017. Among the 1027 sightings, 855 (84%) were of individuals previously documented by Bègue et al.^38^. The total number of individuals observed simultaneously during each dive ranged from 0 to 8 (mean = 1.87 ± 1.30 SD). The relative abundance of tiger sharks per dive had a non-normal, non-homoscedastic distribution (Shapiro–Wilk normality test & Bartlett test of homogeneity of variances, *p*-values < 0.05). The number of bait drums present per dive ranged from 0 to 4 (mean = 1.38 ± 0.80 SD). Bait drum numbers had a non-normal (Shapiro–Wilk normality test, *p*-value < 0.0001) but homoscedastic (Bartlett test of homogeneity of variances, *p*-value = 0.86) distribution. No significant correlation was found between the relative abundance of tiger sharks observed per dive and number of bait drums simultaneously present (Spearman correlation, S = 9.48 × 10^5^, *p*-value = 0.17, rho = 0.10; Supplementary Fig. S1). The relative abundance of tiger sharks observed per dive did not change significantly across the years sampled (Spearman correlation, S = 2.71 × 10^7^, *p*-value = 0.66, rho = 0.019, Supplementary Fig. S2). However, within years the relative abundance of tiger sharks observed per dive was significantly higher during austral winter than austral summer (Kruskal–Wallis rank sum test, $\chi$^2^ = 24.95, df = 1, *p*-value < 0.0001, Supplementary Fig. S3).

### Site fidelity & use

**Figure 1:**
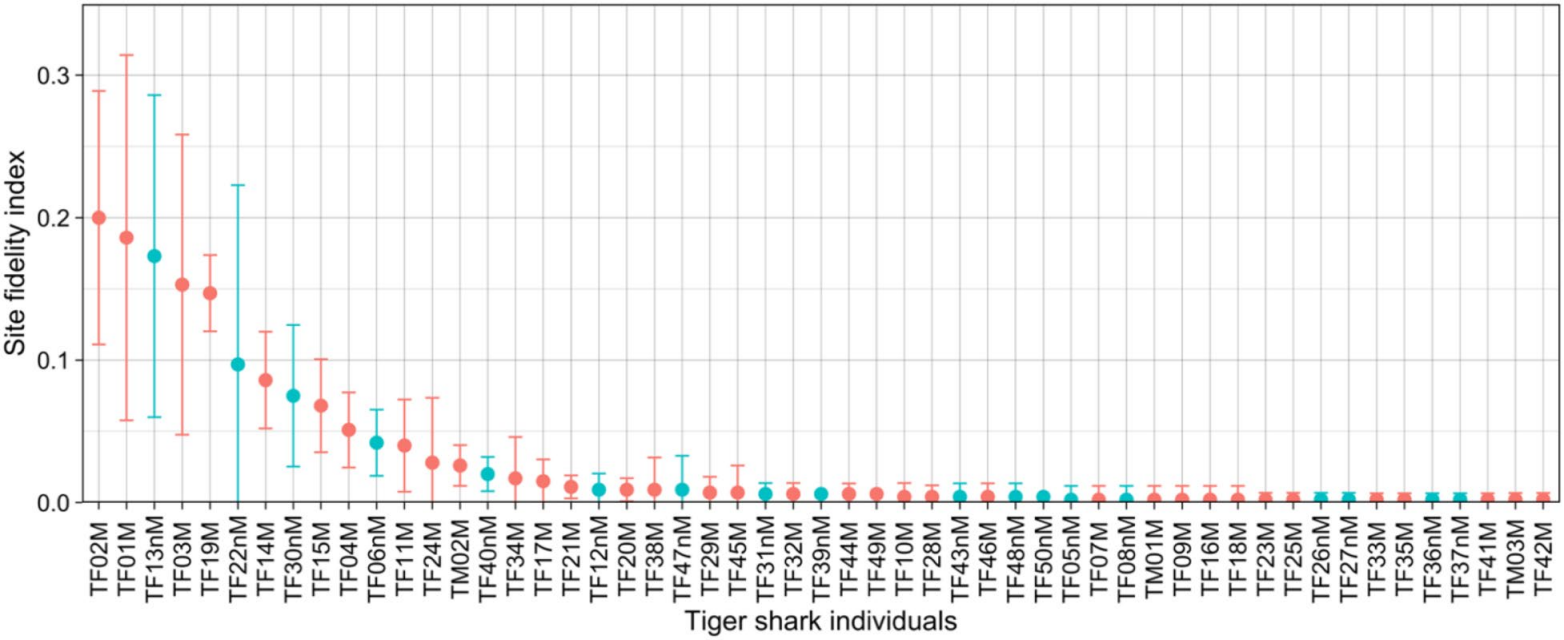
Site Fidelity Indices calculated from sightings of uniquely identifiable G. cuvier at the Vallée Blanche ecotourism site between the 7 October 2012 and the 9 October 2017. Individuals are ordered by decreasing SFI_g_. Standard Deviation (SD) represents variation between years. Mature individuals are represented in red and non-mature in blue.

Tiger sharks (n = 53) observed at the VB site were predominantly (94%) female and primarily sexually mature (64% of females, 100% of males). The sex ratio did not display any significant variation between years (Spearman correlation, S = 2.28 × 10^7^, *p*-value = 0.13, rho = − 0.068) or between seasons (Kruskal–Wallis rank sum test, $\chi$^2^ = 0.52, df = 1, *p*-value = 0.47). The total number of sightings of each individual ranged from 1 to 109 with a mean value of 16.11 ± 28.17 (Table 1). The maximum overall SFI (SFI_g_) value for any photo-identified individual was 0.2 (Fig. 1, Table 1), and the overall mean SFI_g_ value was 0.03 ± 0.05. Five female tiger sharks with SFI_g_ > 0.1 accounted for > 55% of the total sightings at VB despite collectively representing less than 10% of 53 photo-identified tiger sharks (Fig. 1, Table 1). The ten most frequently sighted (SFI_g_ > 0.05) individuals were all females and collectively accounted for more than 76% of the total sightings (Fig. 1, Table 1). Thirty-five (66%) of 53 photo-identified tiger sharks were sighted 5 times or less and 18 (34%) of sharks were sighted only once (Fig. 1, Table 1).

**Table 1:** Site Fidelity Indices calculated from sightings of uniquely identifiable G. cuvier at the Vallée Blanche ecotourism site between the 7 October 2012 and the 9 October 2017.

Shark ID	Date of 1st occurrence	Nb of occurrences	SFIg	SFIa				
				Year 1	Year 2	Year 3	Year 4	Year 5
TF02M	21/10/2012	109	0.200	**0.326**	0.305	0.125	0.168	0.181
TF01M	07/10/2012	101	0.186	**0.413**	0.219	0.227	0.114	0.087
TF03M	18/12/2012	83	0.153	0.152	0.029	0.227	0.074	**0.284**
TF13nM	31/08/2013	94	0.173	0.065	0.0343	0.133	0.087	**0.216**
TF19M	03/11/2013	80	0.147	NA	0.143	0.172	**0.188**	0.129
TF22nM	23/04/2014	53	0.097	NA	**0.248**	0.195	0.013	0
TF14M	05/09/2013	47	0.086	0.022	0.057	0.055	**0.114**	0.078
TF30nM	23/09/2014	41	0.075	NA	0.010	0.078	**0.128**	0.095
TF15M	10/09/2013	37	0.068	0.043	0.029	0.055	**0.114**	0.069
TF04M	14/03/2013	28	0.051	0.043	0.029	0.055	0.034	**0.095**
TF06nM	23/05/2013	23	0.042	0.065	0.048	**0.070**	0.013	0.034
TF11M	10/08/2013	22	0.040	0.065	0	0.055	0.020	**0.078**
TF24M	29/06/2014	15	0.028	NA	0.010	0	**0.094**	0
TM02M	22/08/2013	14	0.026	**0.043**	0.019	0.039	0.027	0.009
TF40nM	19/05/2015	11	0.020	NA	NA	0.023	**0.040**	0.017
TF34M	13/11/2014	9	0.017	NA	NA	0.008	**0.054**	0
TF17M	24/09/2013	8	0.015	0.022	0	0.023	0	**0.034**
TF21M	20/12/2013	6	0.011	NA	**0.019**	0	0.013	0.017
TF12nM	29/08/2013	5	0.009	0.022	0	**0.023**	0	0.009
TF20M	21/11/2013	5	0.009	NA	**0.019**	0.008	0.013	0
TF38M	14/04/2015	5	0.009	NA	NA	**0.039**	0	0
TF47nM	20/07/2016	5	0.009	NA	NA	NA	**0.034**	0
TF29M	12/09/2014	4	0.007	NA	0.010	**0.023**	0	0
TF45M	02/06/2016	4	0.007	NA	NA	NA	**0.027**	0
TF31nM	30/09/2014	3	0.006	NA	0.010	**0.016**	0	0
TF32M	23/10/2014	3	0.006	NA	NA	**0.016**	0	0.009
TF39nM	21/04/2015	3	0.006	NA	NA	0.008	0.007	**0.009**
TF44M	24/05/2016	3	0.006	NA	NA	NA	0.007	**0.017**
TF49M	14/08/2017	3	0.006	NA	NA	NA	NA	**0.026**
TF10M	23/07/2013	2	0.004	**0.022**	0	0.008	0	0
TF28M	31/08/2014	2	0.004	NA	**0.019**	0	0	0
TF43nM	05/04/2016	2	0.004	NA	NA	NA	**0.013**	0
TF46M	15/07/2016	2	0.004	NA	NA	NA	**0.013**	0
TF48nM	18/08/2016	2	0.004	NA	NA	NA	**0.013**	0
TF50nM	09/09/2017	2	0.004	NA	NA	NA	NA	**0.017**
TF05nM	11/04/2013	1	0.002	**0.022**	0	0	0	0
TF07M	23/05/2013	1	0.002	**0.022**	0	0	0	0
TF08nM	04/07/2013	1	0.002	**0.022**	0	0	0	0
TM01M	04/07/2013	1	0.002	**0.022**	0	0	0	0
TF09M	23/07/2013	1	0.002	**0.022**	0	0	0	0
TF16M	18/09/2013	1	0.002	**0.022**	0	0	0	0
TF18M	03/10/2013	1	0.002	**0.022**	0	0	0	0
TF23M	15/05/2014	1	0.002	NA	0.010	0	0	0
TF25M	08/07/2014	1	0.002	NA	**0.010**	0	0	0
TF26nM	10/07/2014	1	0.002	NA	**0.010**	0	0	0
TF27nM	28/08/2014	1	0.002	NA	**0.010**	0	0	0
TF33M	03/11/2014	1	0.002	NA	NA	**0.008**	0	0
TF35M	27/11/2014	1	0.002	NA	NA	**0.008**	0	0
TF36nM	26/02/2015	1	0.002	NA	NA	**0.008**	0	0
TF37nM	09/04/2015	1	0.002	NA	NA	**0.008**	0	0
TF41M	24/09/2015	1	0.002	NA	NA	**0.008**	0	0
TM03M	29/10/2015	1	0.002	NA	NA	NA	**0.007**	0
TF42M	25/11/2015	1	0.002	NA	NA	NA	**0.007**	0

Bold values indicate the year of maximum SFI_a_ for the each individual. Individuals are ordered by decreasing SFI_g_.

Individual annual site fidelity (SFI_a_) varied with no consistent patterns evident across years (Spearman correlation, S = 2.15 × 10^6^, *p*-value = 0.76, rho = − 0.020, Supplementary Fig. S4) or between individuals (Kruskal–Wallis rank sum test, $\chi$^2^ = 134.14, df = 52, *p*-value = 3.48 × 10^−9^, no pairwise comparisons were significant with Dunn’s test, Supplementary Fig. S5). Some tiger sharks were sighted during every year of the 6-year survey whereas others were absent for up to a year between sightings (Fig. 1, Table 1). The maximum annual SFI_a_ value was 0.413, obtained for TF01M during the first year of study, and the minimum annual SFI value was 0. The 10 most frequently sighted individuals (SFI_g_ > 0.05) did not show any significant variation in SFI_a_ between years (Spearman correlation, S = 1.61 × 10^4^, *p*-value = 0.66, rho = 0.067, Supplementary Fig. S6).

**Figure 2:**
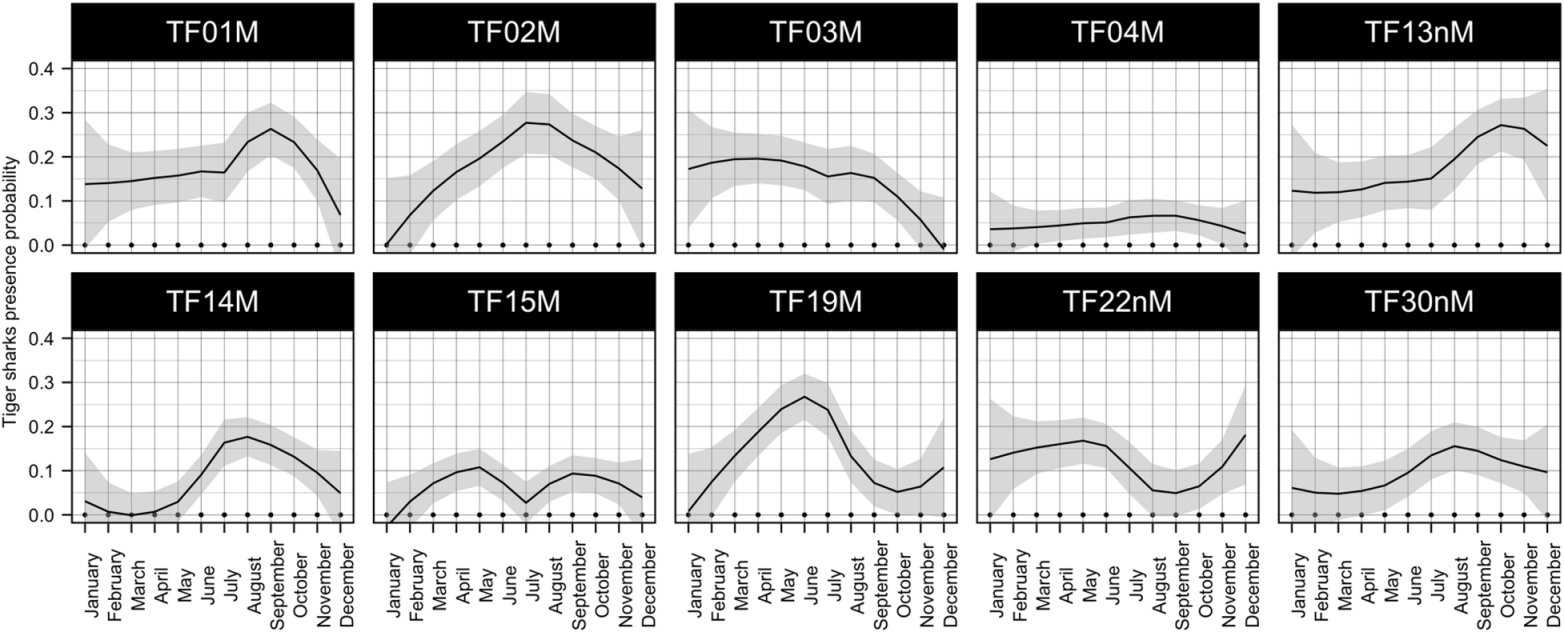
Inter-individual differences in seasonal probability of presence for the ten most frequently sighted (SFI_g_ > 0.05) tiger sharks. Shaded areas represent 95% confidence intervals.

**Figure 3:**
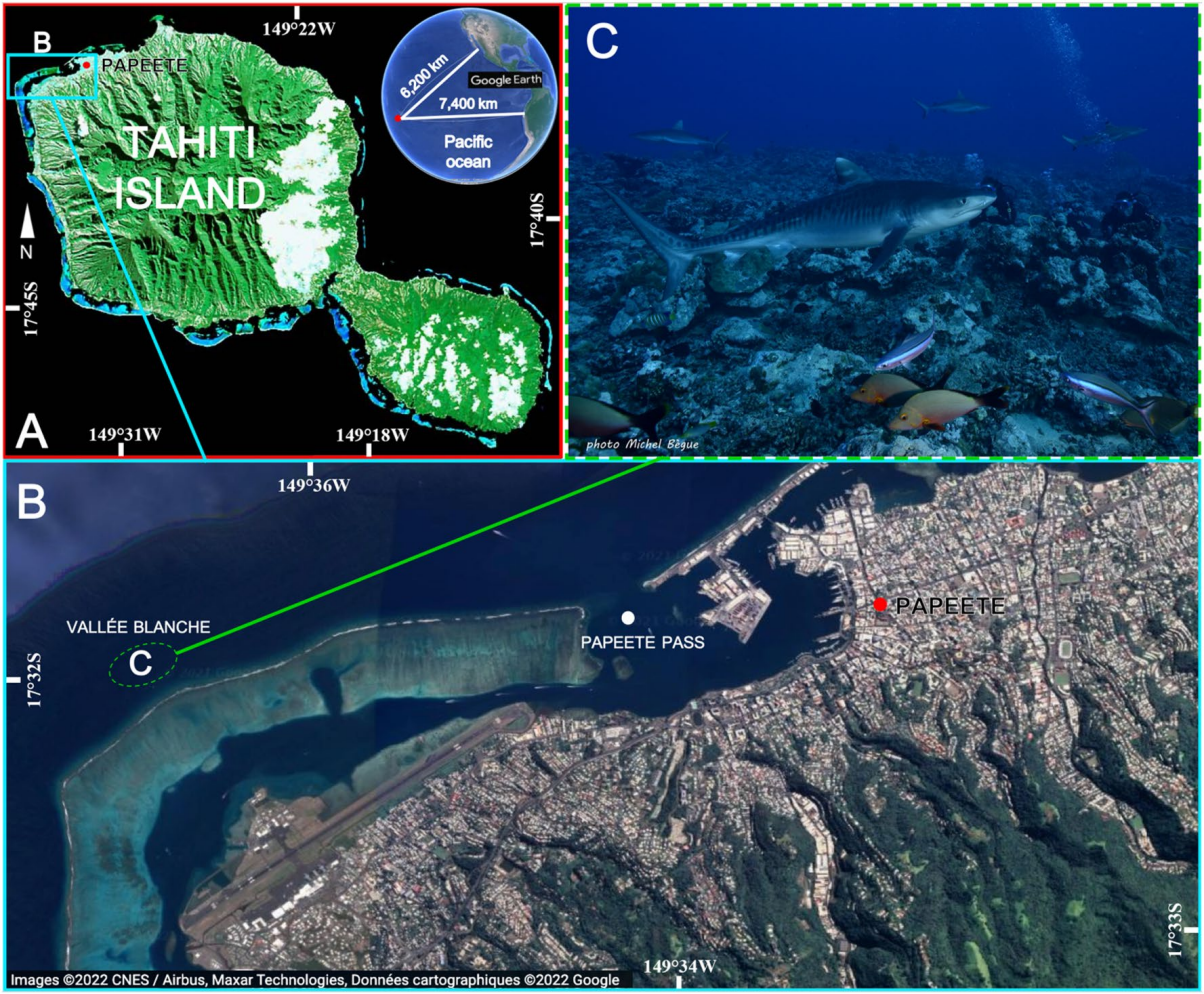
Map of the study area with (**A**) Its location off Tahiti, French Polynesia; (**B**) Its satellite view (Google Earth); (**C**) An underwater view of shark provisioning (photo credit: Michel Bègue). White circle: location of the Papeete pass, when red circle represents the city of Papeete, capital of Tahiti island.

Despite a general trend of more frequent tiger shark sightings during the austral summer, some intra-specific variation in seasonal patterns of tiger shark sightings was also evident (Fig. 3). For example, sightings of 8 of the 10 most frequently sighted tiger sharks (SFI_g_ > 0.05, 28–109 occurrences), peaked during austral summer and were lowest during austral winter, whereas TF03M was present most frequently between January and September and TF22nM between December and June (Table 1, Fig. 3). All sharks except TF04M showed a strong intra-individual variability in presence pattern depending on the month of the year. Furthermore, 7 sharks were rarely present (presence probability < 0.05) or completely absent from the ecotourism site for several months. Other individuals (e.g. TF04M) showed less seasonal variation in presence, or had a high year round probability of presence (e.g. TF13nM) (Fig. 2). No significant difference in presence between the number of non-mature and mature individuals was observed between austral winter and austral summer ($\chi$^2^ test: $\chi$^2^ = 0.32, df = 1, *p*-value = 0.57).

## Discussion

We found no evidence of either increasing site fidelity or established resident behavior (SFI > 0.5)^39^ by tiger sharks at the VB ecotourism site despite over 6 years of regular provisioning at this location. Overall site fidelity (SFI_g_) of each of the 53 uniquely identified tiger sharks was low (⩽ 0.2) and the maximum annual site fidelity (SFI_a_) for any individual was 0.413 and declined over time. Furthermore, no significant rise in the number of tiger sharks sighted simultaneously during each dive was apparent. This observed lack of significant impact of regular provisioning on tiger shark fidelity in French Polynesia is consistent with the results of previous studies on highly migratory sharks including tiger sharks in the Caribbean^25^, white sharks *Carcharodon carcharias* at cage diving sites in South Africa^40^ and Mexico^41,42^, and bull sharks *Carcharhinus leucas* in Fiji^24^, none of which found any demonstrable influence of provisioning on site fidelity. By contrast, previous studies of less mobile species such as sicklefin lemon sharks *Negaprion acutidens*^16,23^ and blacktip reef sharks *Carcharhinus melanopterus* (Quoy & Gaimard 1824)^19^ did detect increasing fidelity over time at other Polynesian provisioning sites. These interspecific differences in susceptibility to provisioning effects on fidelity may stem from fundamental differences in the spatial ecology of these various species. Species displaying naturally high levels of mobility and strong variability in movement patterns (e.g. tiger sharks) may simply be harder to retain at provisioning sites than highly reef-associated sharks (e.g. blacktip reef sharks).

Inter-individual differences in tiger shark SFI_g_ observed at VB may also simply reflect natural variability in movement patterns and home range locations. Previous telemetry studies in Hawaii have shown tiger sharks occupy core-structured home ranges around oceanic islands^15,28,30,43,44^. Although their entire home range may span multiple oceanic islands and extend into open-ocean, individual tiger sharks spend most of their time within a smaller Core Use Area (CUA) often associated with one stretch of coastline, and multiple individuals routinely occupy highly overlapping CUAs within the same general area^28^. Similar tiger shark home range characteristics may exist around the oceanic Windward Islands of French Polynesia. Thus, the most frequently sighted tiger sharks in this current study may have CUAs that include VB but still exhibited overall low fidelity at the ecotourism site because they continually roam back and forth within a CUA that may be as large as the entire North coast of Tahiti, similar to the observations made in Hawaii’s oceanic islands^28^. Conversely, tiger sharks that were only occasionally sighted may occupy CUAs that do not include VB and their sporadic appearances at the ecotourism site reflect less-frequent wider-ranging movements outside their CUAs. Thus, the observed patterns of tiger shark sightings at provisioning ecotourism sites could be entirely explained by natural home range structure and there is no evidence that provisioning is significantly modifying their movement patterns or CUA locations. Satellite tagging could be used to empirically define tiger shark CUAs around Tahiti island to evaluate this hypothesis.

Although home range structure alone could reasonably explain observed SFI patterns it is important to consider a potential confounding effect of individual shark “boldness” on those patterns. We cannot rule out the possibility that bolder sharks willing to come closer to divers were seen most frequently whereas other more timid tiger sharks were also attracted to the site by provisioning activities but simply remained out of sight for most of the time. Previous studies with juvenile bull sharks showed some individuals being detected in riskier locations more often than others^45^ reinforcing the possibility that variable boldness among individuals could be contributing to the observed patterns of tiger shark use of the VB site. Boldness can be related to body size in sharks with larger animals generally dominant at feeding aggregations and smaller sharks excluded until larger individuals are satiated^46,47^. However, at the VB site three subadult tiger sharks were among the 10 most frequently sighted individuals and overlapped in presence with large adults indicating that body size alone does not explain observed SFI patterns. Additional telemetry studies of tiger shark movements around Tahiti could help to clarify whether individual boldness influences diver-based surveys of tiger shark presence. Furthermore, a recent study suggests that individual tiger sharks displaying high levels of testosterone or plasma steroids may be correlated with longer time spent at the provisioning site^48^. Thus, impact of physiological state on site occupancy must be further explored in future studies.

The significant and consistent seasonal fluctuations in tiger shark abundance at the VB ecotourism site are a further indication that their natural movement patterns are not significantly influenced by provisioning. The distinctive high-low winter-summer pattern of tiger shark abundance at VB is consistent with seasonal migrations of the kind previously documented in tiger sharks in other locations without artificial provisioning^29,33^, and also in tiger sharks and other species at other provisioning ecotourism sites^15,22–24,49,50^. The drivers of these seasonal movements could include reproduction or seasonal prey abundance. For example, the VB site is predominantly frequented by adult females, similar to Tiger Beach in Bahamas^51^, which may migrate seasonally for mating or pupping purposes. Seasonal variations in tiger shark abundance in other areas have been related to foraging on seasonal prey-aggregations such as fledging albatross *Phoebastria spp.* in Hawaii^30^ or loggerhead turtles *Caretta caretta* (Linnaeus 1758)^52^ in the North Atlantic ocean. Although the drivers of seasonal cycles of tiger shark abundance at the VB site are unknown, their continued manifestation despite consistent provisioning demonstrates that artificial feeding does not overwhelm these natural patterns.

Wildlife provisioning is controversial largely because it can be deleterious to animals and people^53^. Examples of wildlife provisioning leading to negative outcomes include the death of a 9-year old boy resulting in the culling of 31 Australian dingoes *Canis lupus dingo* (Linnaeus 1758) in the Fraser Island National Park^54^, a dangerous increase in the number of Indonesian Komodo dragons *Varanus komodoensis* (Ouwens 1912) in provisioning areas^55^, and provisioned Thai macaques *Macaca fascicularis* (Raffles 1821) showing more aggressive behaviours than non-provisioned individuals towards humans^56^. Thus, potential impacts of shark provisioning must be carefully considered in order to avoid negative consequences to sharks and people. Our current results support those of other recent studies suggesting a general lack of chronic or irreversible impacts of feeding activities on sharks^24,25,40,50,57–59^. In fact the positive experiences of participants in these activities leads to important economic benefits and increased support for shark conservation^60–62^. However, careful management and adherence a strict code of conduct are essential to keep shark provisioning ecotourism safe and ecologically sustainable^10,13,16,58,63,64^. For example, regulations restricting shark provisioning permits to certified operators, might be a way to penalize non-compliance with management plans. Such measures are intended to increase the safety of participants and target species and are already successfully applied in Australia with the endangered whale shark *Rhincodon typus* (Smith 1828) as well as with the critically endangered sand tiger shark *Carcharias taurus* (Rafinesque 1810)^65–67^. Our results suggest that provisioning ecotourism at the VB site did not significantly influence tiger shark site fidelity and we suggest that this practice can be kept safe and sustainable through appropriate regulations including prohibiting risky practices such as hand feeding^16,63^.

## Methods

### Study area

The study was carried out at the “Vallée Blanche” (VB) dive site (S 17.542°; W 149.624°), located on the outer slope of the barrier reef close to the Papeete pass on North-West coast of Tahiti (French Polynesia) (Fig. 3A,B). This site covers an area of approximately 40,000 m^2^ characterized by a central valley of sand and coral debris at a depth of between 15 and 20 m, surrounded by pinnacles of hard corals, and adjacent to a steep drop-off and exposed to strong and variable currents^38^. Diving centers initiated shark feeding activity in 2011 in order to concentrate sharks in a particular area and to increase the probabilities of sighting six different species: blacktip reef sharks *Carcharhinus melanopterus*, grey reef sharks *Carcharhinus amblyrhynchos* (Bleeker 1856), whitetip reef sharks *Triaenodon obesus* (Rüppel 1837), tawny nurse sharks *Nebrius ferrugineus* (Lesson 1831), sicklefin lemon sharks *Negaprion acutidens* and tiger sharks *Galeocerdo cuvier* (Fig. 3C). Overall, 53 tiger sharks (50 females and 3 males) were individually photo-identified using unique characteristics, including skin pattern such as stripes or deep scars, wounds, or damaged dorsal or pectoral fins^38,47^. Individual identities were carefully validated via a thorough cross comparison of different body areas.

### Data collection

Shark feeding activity occured year-round at VB, with almost daily provisioning sessions during Polynesian austral winter (May to October) but more sporadic provisioning during austral summer due to frequently unfavorable ocean conditions (high swell) and fewer tourists, but did occur whenever sea conditions were suitable. Sharks were attracted to the site using 4–6 yellowfin tuna heads *Thunnus albacares* (Bonnaterre 1788) contained in metal washing-machine drums (to prevent bait consumption) placed on the seabed at a depth of 18 m. The attraction process began each day at 8 AM on the first dive of the day. A second dive usually took place at 11 AM without food release, followed by a third dive at 2 PM, where the bait was released (head by head) at the end of the dive and fed to the waiting sharks. This schedule was based on the typical activity schedules of local dive centers. Dives were typically 45 min in duration. During the sampling period, VB was visited by 10 dive centers of which up to 4 attracted sharks at the same time with multiple bait drums distributed across the dive spot^68^. There was no coordination of these activities among dive operators but all dive centers involved in provisioning sharks visited VB almost daily for at least one dive. Due to the difficulty in evaluating the exact amount of tuna heads provided when several diving operators were sharing VB, the bait quantity was evaluated using the number of visible drums present. All dives were considered as independent in the following analysis.

Relative abundance, defined as the number of tiger sharks sighted per dive, identity, and size were recorded by an expert diver (M. Bègue) with more than 500 dives on the site. All the sharks identified in a previously developed database, were individually recognizable by based on their unique markings^38^. The size was assessed visually by the expert, and confirmed with the use of stereo-laser photogrammetry on several animals (n = 12), which showed an acceptable error < 5% of the estimated size. This non-invasive technique consists of two perfectly parallel laser dots calibrated at a specific distance apart, allowing the total length of the animal to be calculated from pictures^69,70^. ImageJ was used to compare the number of pixels between the two dots and the number of pixels for the total length of the shark. The sexual maturity status of each individual was estimated from their total lengths, with females > 3.30 m TL and males > 2.92 m TL considered to be sexually mature^71^. Each individual was assigned an alphanumeric identification code consisting of “T” for the species (Tiger), “F” (Female) or “M” (Male) for the sex, a two-digit number linked to the ranking of the first observation at VB (first tiger observed = 01, second observed = 02 etc.), and “M” (Mature) or “nM” (non-Mature) indicating the sexual maturity of the animals.

### Evaluation of potential deleterious effects of provisioning

Data analyses were conducted in R (V 4.2.2)^72^ with the significance level set to 0.05. Shapiro–Wilk tests were used to check the normality and Bartlett tests to check the homoscedasticity of the distributions of different variables studied, resulting in non-parametric tests being selected for the data analyses. We used a Spearman correlation test to compare baiting level, quantified as the number of drums simultaneously present during the dive, with tiger shark relative abundance. We used Kruskal–Wallis rank sum tests to evaluate yearly and seasonal variations in tiger shark relative abundance with relative abundance as response variable and the year of sampling or the season as potential explanatory variables. Wilcoxon tests were used to make pairwise comparisons of significant results in order to identify periods of the year where tiger sharks were sighted significantly more frequently by divers at the VB site.

We calculated the global site fidelity index (SFI_g_) and the annual site fidelity index (SFI_a_) for each photo-identified individual by dividing the number of dives where the shark was seen by the total number of dives conducted during the respective sampling period. SFI values range from 0 to 1, with low values characterizing tiger sharks with a low site fidelity at VB and vice versa. An individual was considered as “resident” for a given year if the calculated SFI_a_ was equal to or higher than 0.5, and as “inter-annual resident” when it showed a SFI_a_ equal or higher than 0.5 over consecutive years^40^. To explore potential intra-specific differences in site use during the sampling period, we used Durbin Watson tests to examine the autocorrelation in sightings of individuals with > 25 total observations during the 5 years of sampling. For the 10 most-frequently sighted photo-identified tiger sharks, we generated response curves of monthly probability of presence deduced from the binomial presence/absence (1/0) data and fitted with a Loess smoother. $\chi$^2^ tests were used to evaluate whether presence patterns of the most frequently sighted sharks was significantly associated with either maturity status (mature and non-mature) or season (austral summer or austral winter). If significant, post hoc analysis based on residuals of Pearson’s $\chi$^2^ test for count data were used to further characterize the relationships. For the 10 most-frequently sighted tiger sharks, we used Kruskal–Wallis tests to evaluate the influence of study year on SFI_a_. Significant results were further assesses using Wilcoxon tests paiwise comparisons.

## Supplementary Information

Supplementary Figures.

## Data Availability

The datasets used and analyzed during the current study are available from the corresponding author upon reasonable request.
