# Supplementary material for: Provisioning ecotourism does not increase tiger shark site fidelity
Source: Sci Rep. 2023 May 13;13:7785. doi: 10.1038/s41598-023-34446-8 (PMC10183044; doi:10.1038/s41598-023-34446-8)
Supplement: Supplementary file 1 — Supplementary Figures. [file 41598_2023_34446_MOESM1_ESM.docx]

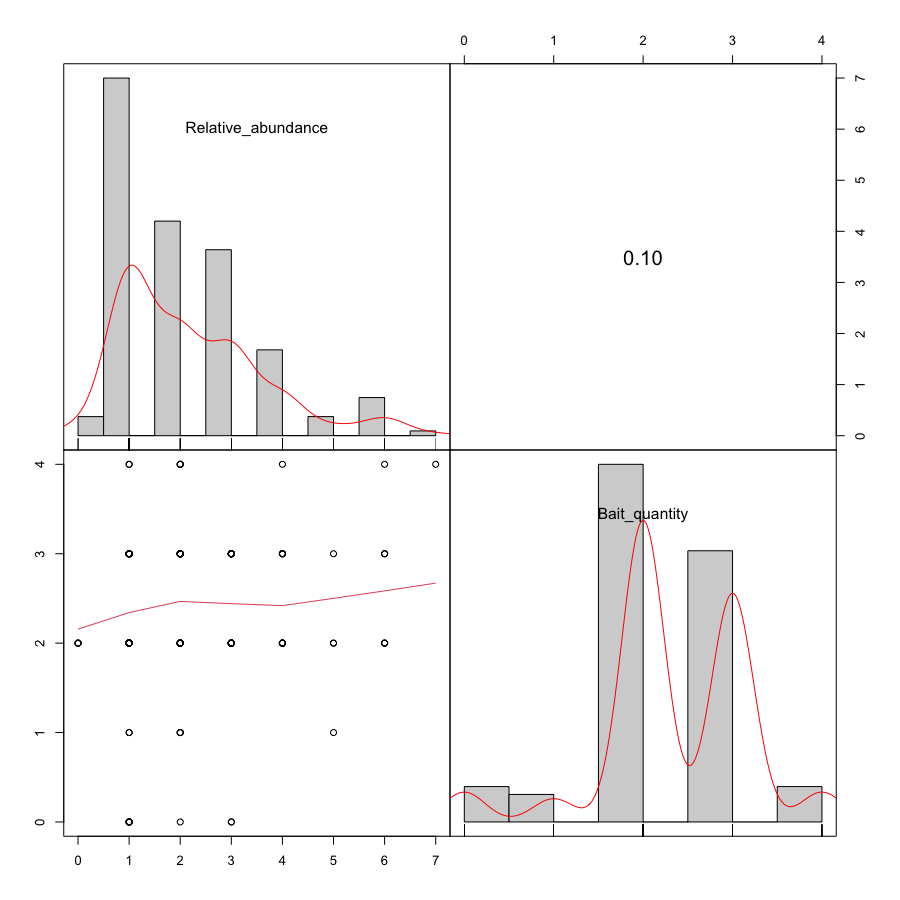

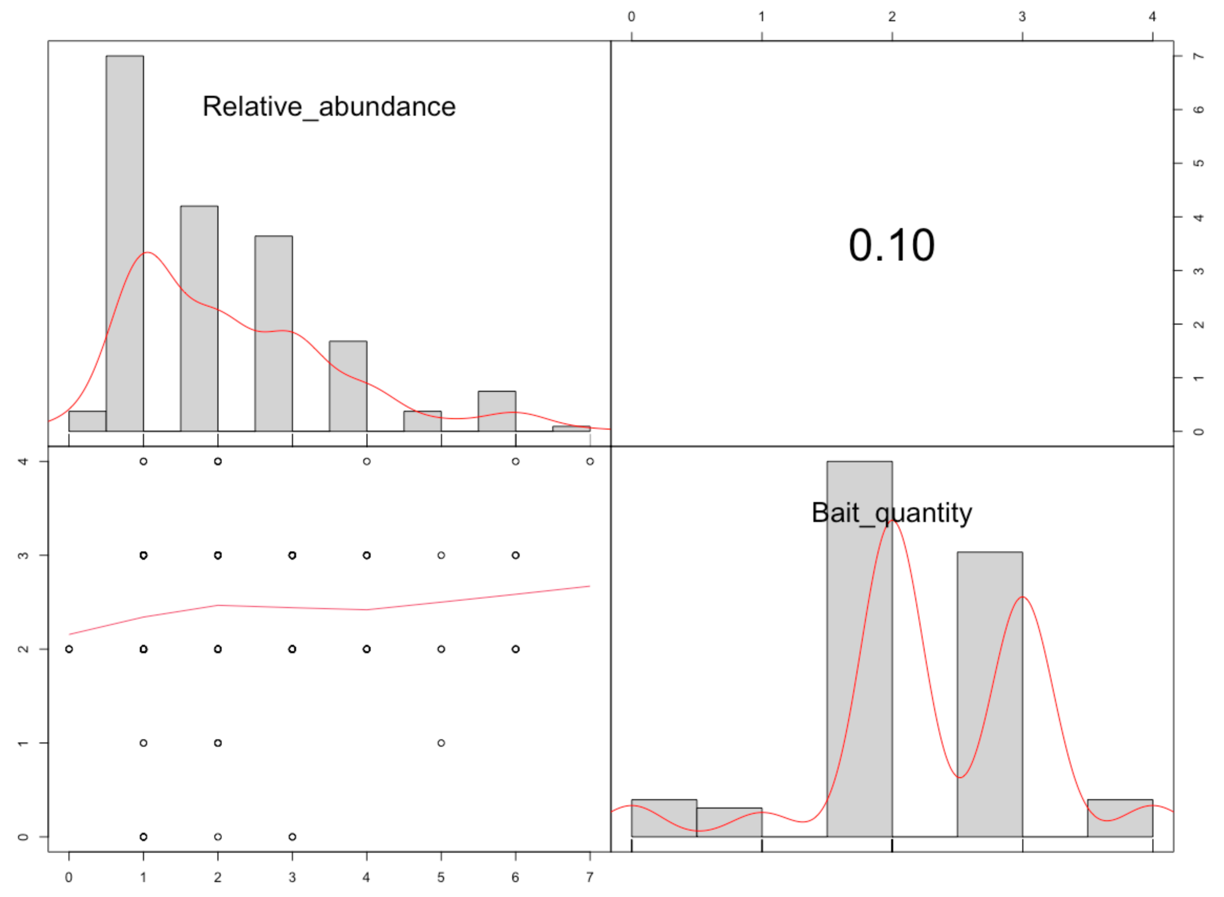
Supplementary Fig. S1: Visualization of the correlation between the relative abundance of tiger sharks and the quantity of drums at the provisioning site. The value on the top right is the value of the Spearman’s correlation. The bivariate scatterplots with a fitted line are on the bottom left. Both display an absence of correlation between these two variables.


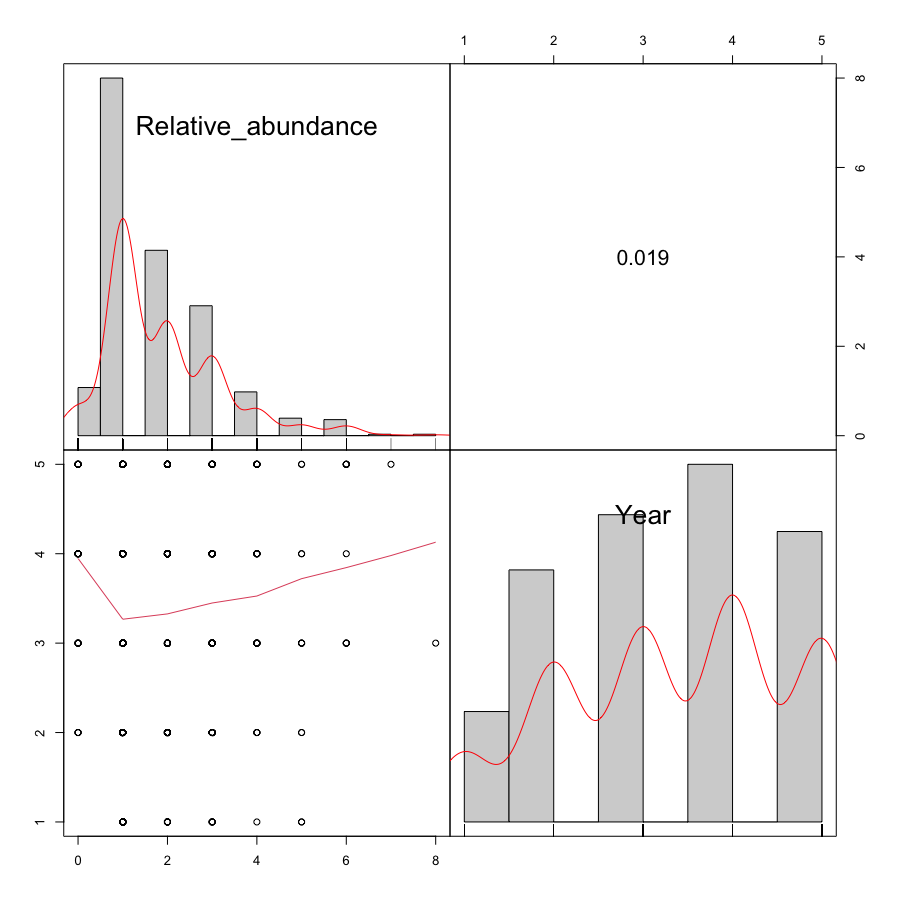
Supplementary Fig. S2: Visualization of the correlation between the relative abundance of tiger sharks and the year sampled. The value on the top right is the value of the Spearman’s correlation. The bivariate scatterplots with a fitted line are on the bottom left. Both display an absence of correlation between these two variables.


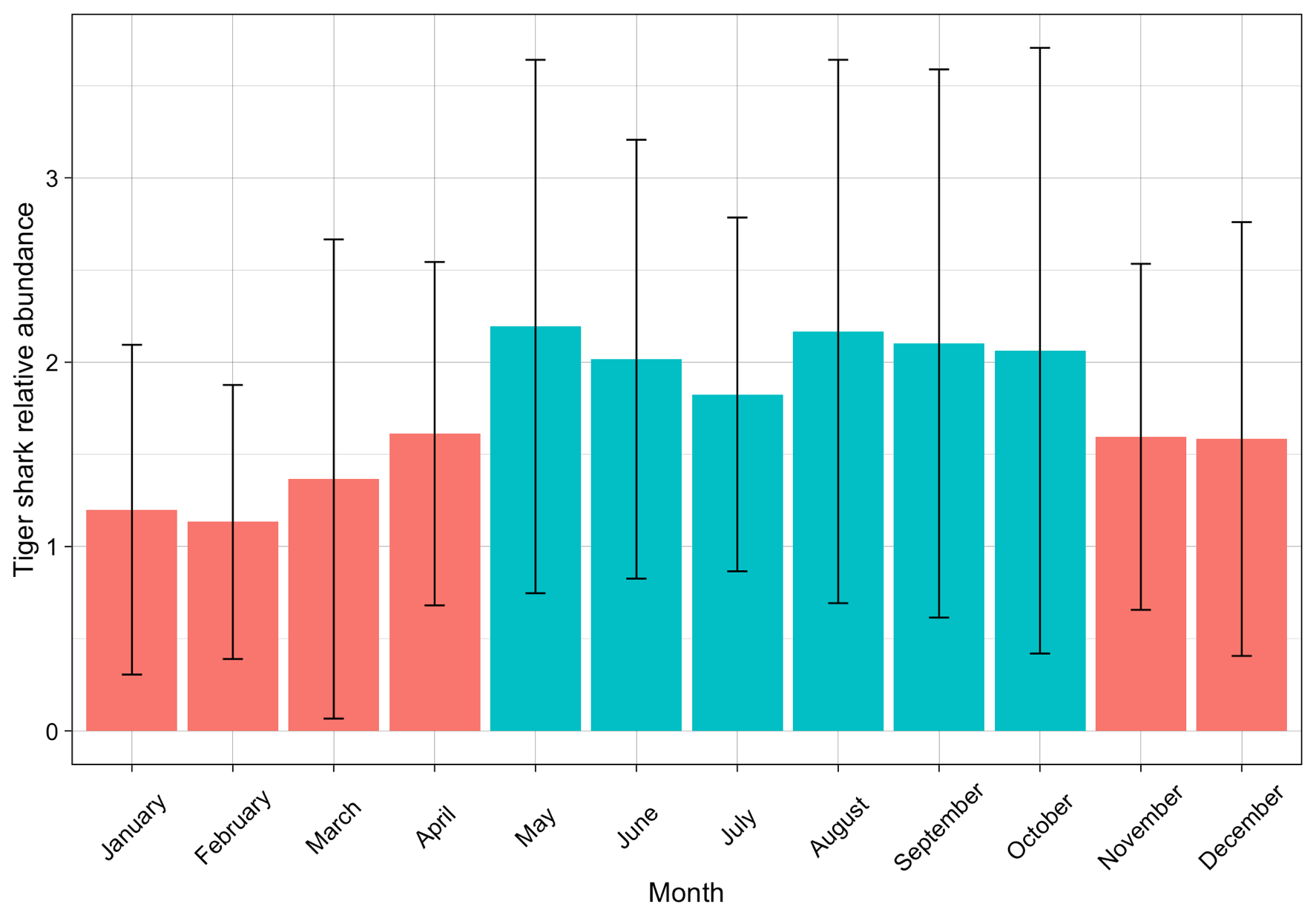


Supplementary Fig. S3: Seasonal variation in tiger shark relative abundance. Red shading: Austral summer months. Blue shading: Austral winter months. Standard Deviation (SD) represents variation between years.


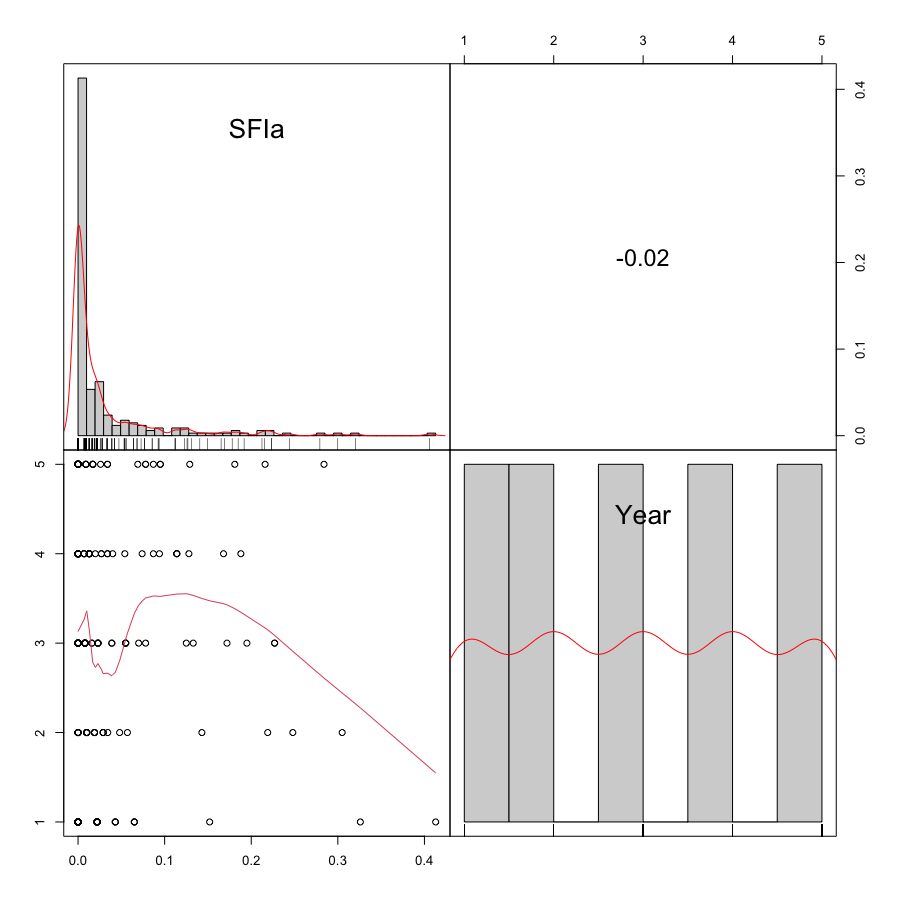


Supplementary Fig. S4: Visualization of the correlation between the SFI_a_ and the year sampled. The value on the top right is the value of the Spearman’s correlation. The bivariate scatterplots with a fitted line are on the bottom left. Both display an absence of correlation between these two variables.


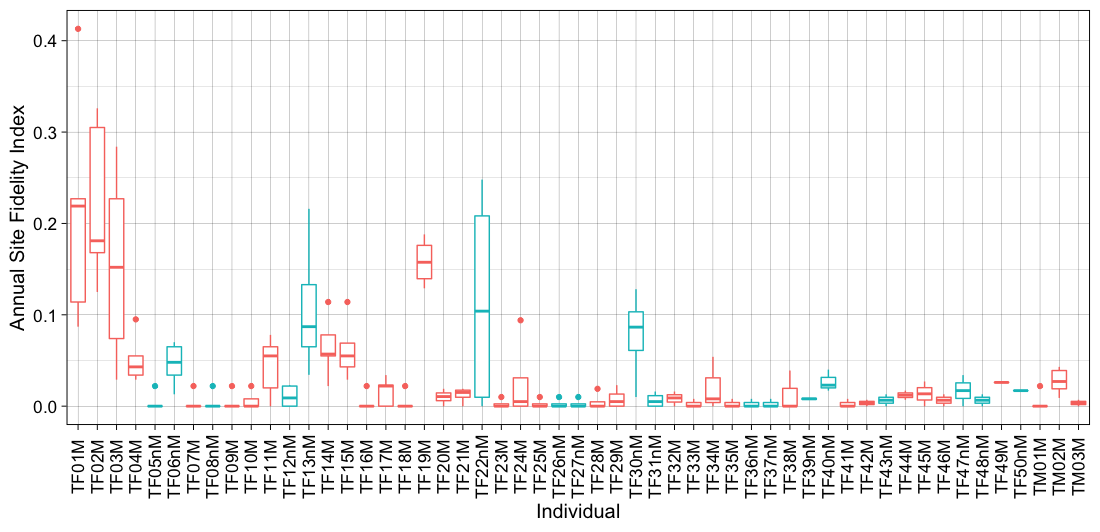
Supplementary Fig. S5: Variation in SFI_a_ between individual photo-identified tiger sharks. Mature individuals are represented in red and immature in blue.


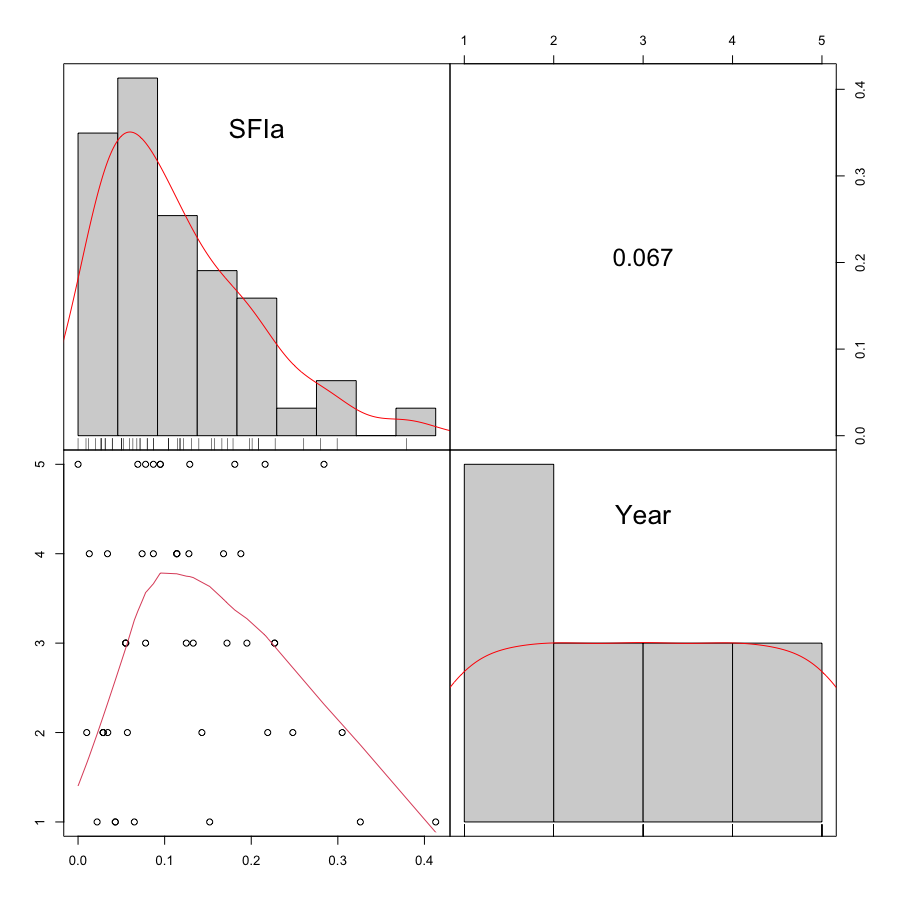
Supplementary Fig. S6: Visualization of the correlation between the SFI_a_ and the year sampled for the 10 most frequently sighted tiger sharks. The value on the top right is the value of the Spearman’s correlation. The bivariate scatterplots with a fitted line are on the bottom left. Both display an absence of correlation between these two variables.
